# Supplementary material for: Cyclic-di-GMP signalling and biofilm-related properties of the Shiga toxin-producing 2011 German outbreak Escherichia coli O104:H4
Source: EMBO Mol Med. 2014 Oct 31;6(12):1622–37. doi: 10.15252/emmm.201404309 (PMC4287979; doi:10.15252/emmm.201404309)
Supplement: Supplementary file 2 — Supplementary Figure S2 [file emmm0006-1622-sd2.pdf]

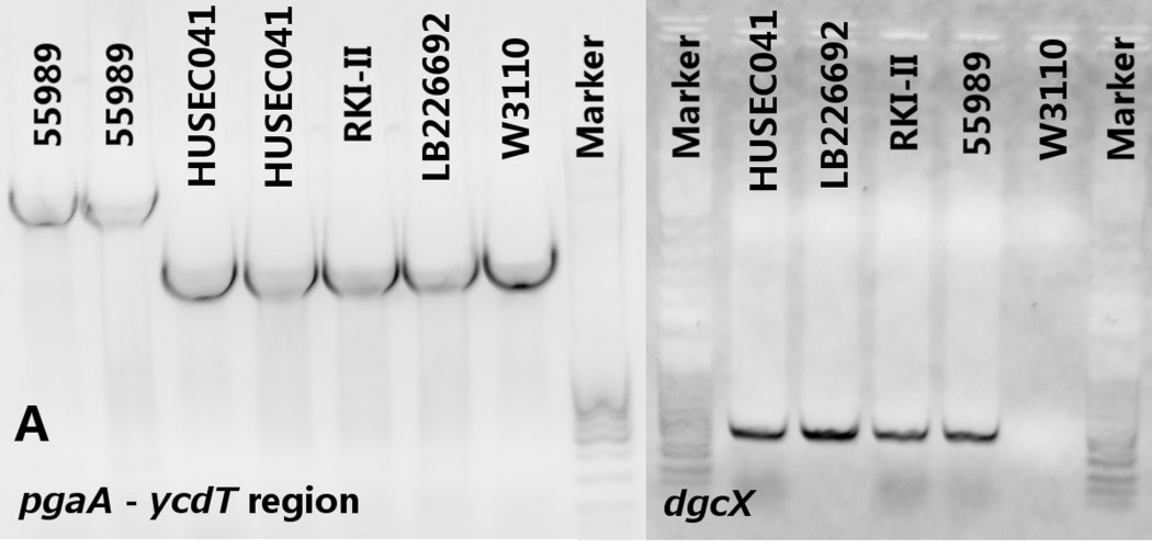

B **W3110, LB226692, HUSEC041:**

```
                                                   -35
ATCAGGACTTTCGAAAAATCCGAAATCATGCATCGGAATTTACTGATTTA
  (18bp)            -10           +1
ATTATTTTAATCCTAATTTATTTTGAAAAAGGCATTGGGA-------ATG
                                    (5´-UTR=235bp)
```

55989:

```
          -35         (18bp)          -10          +1   -35
AAGCCACTGGAGCACCTCAAAAACACCATCATACACTAAATCAGTAAGTT
          (17bp)          -10          +1
GGCAGCATCATCCTAATTTATTTTGAAAAAGGCATTGGGA-------ATG
                                    (5´-UTR=231bp)
```
