# Supplementary figures and images for: Cyclic-di-GMP signalling and biofilm-related properties of the Shiga toxin-producing 2011 German outbreak Escherichia coli O104:H4
Source: EMBO Mol Med. 2014 Oct 31;6(12):1622–37. doi: 10.15252/emmm.201404309 (PMC4287979; doi:10.15252/emmm.201404309)

**A**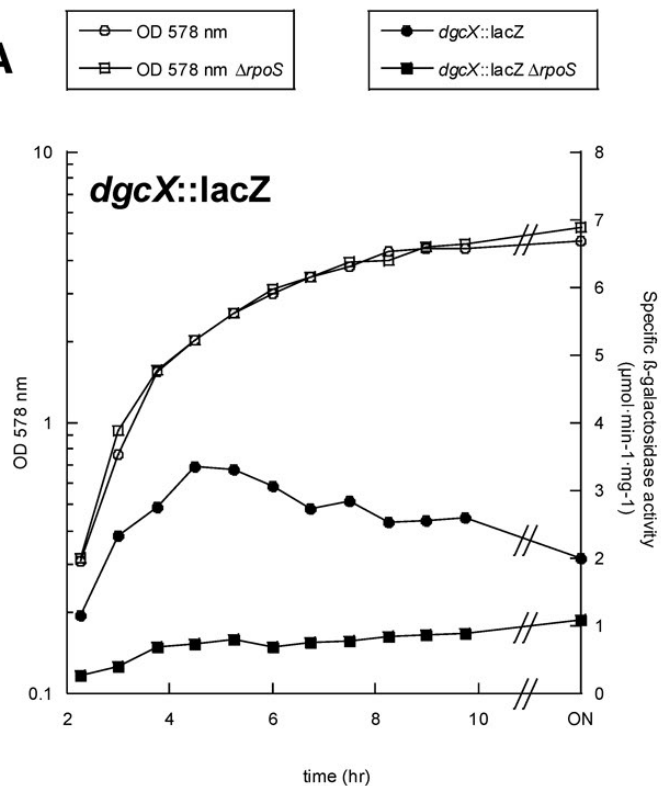**B**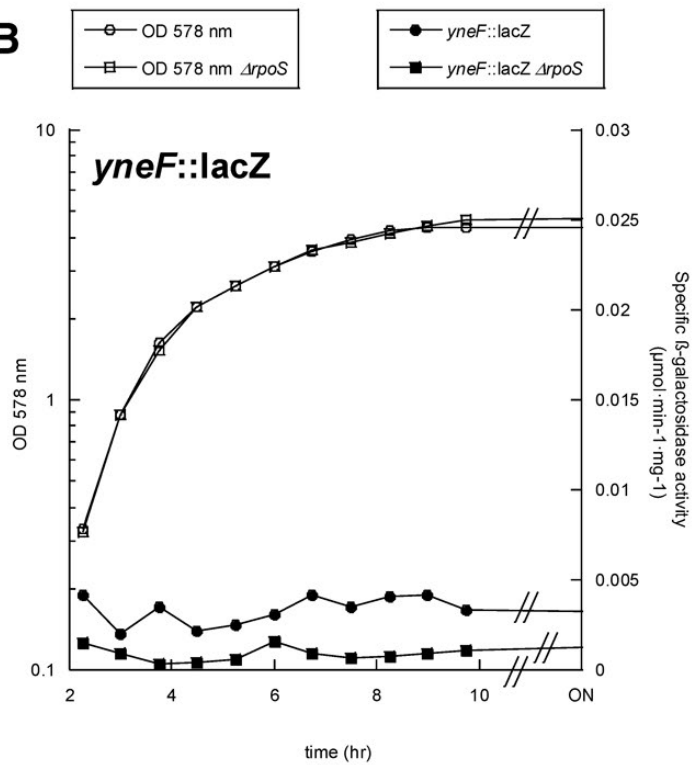

Supplement: Supplementary file 3 — Supplementary Figure S3 [file emmm0006-1622-sd3.pdf]

EDL933

EAEc 55989

HUSEC041

RKI II-2027

K-12 W3110

LB 28°C

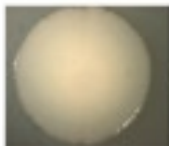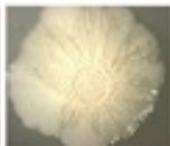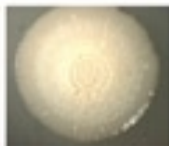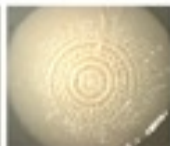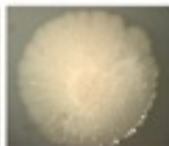

LBnoS 28°C

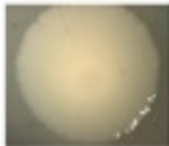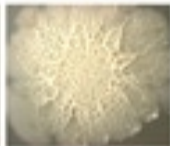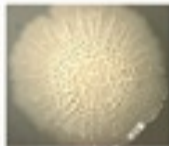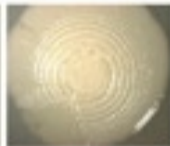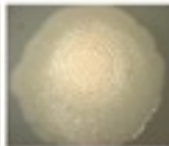

LB 37°C

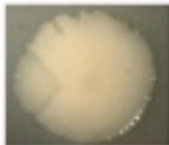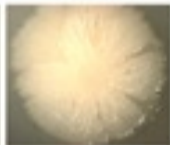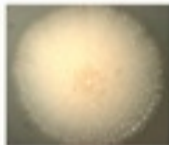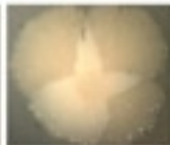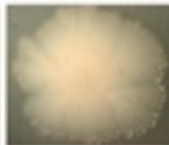

LBnoS 37°C

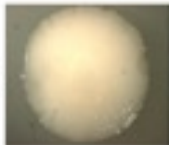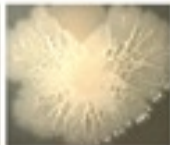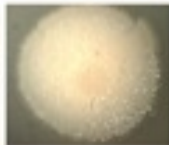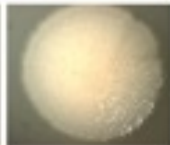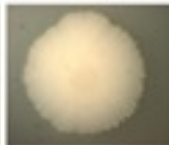

Supplement: Supplementary file 4 — Supplementary Figure S4 [file emmm0006-1622-sd4.pdf]

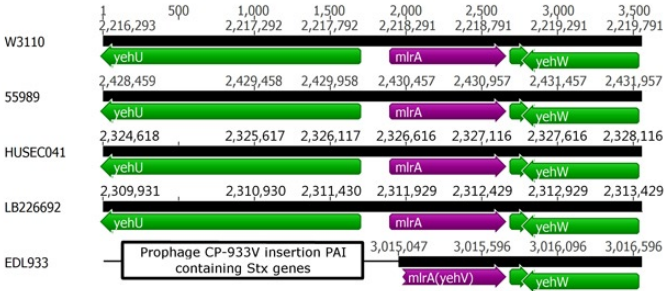

Supplement: Supplementary file 5 — Supplementary Figure S5 [file emmm0006-1622-sd5.pdf]

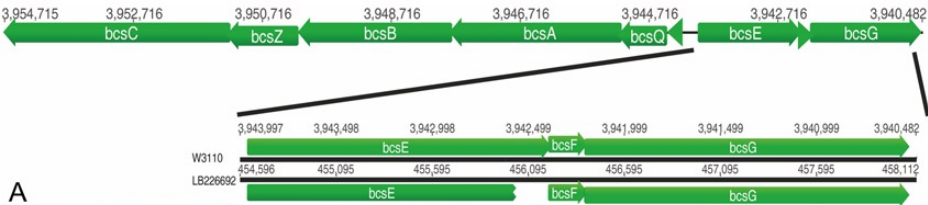

A

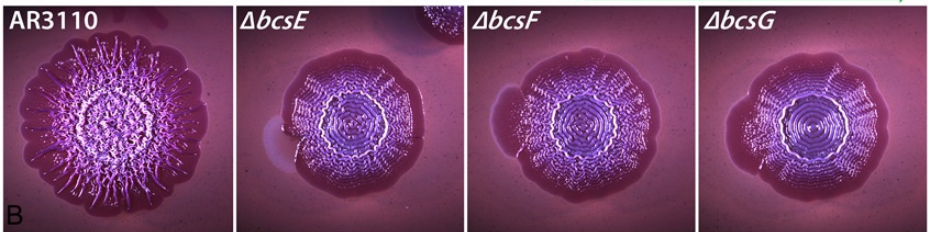

Supplement: Supplementary file 6 — Supplementary Figure S6 [file emmm0006-1622-sd6.pdf]
