# Supplementary material for: Cyclic-di-GMP signalling and biofilm-related properties of the Shiga toxin-producing 2011 German outbreak Escherichia coli O104:H4
Source: EMBO Mol Med. 2014 Oct 31;6(12):1622–37. doi: 10.15252/emmm.201404309 (PMC4287979; doi:10.15252/emmm.201404309)
Supplement: Supplementary file 7 — Supplementary Table S1 [file emmm0006-1622-sd7.pdf]

Gene	Nucl.	W3110	55989	HUSEC041	LB226692	EDL933	Remarks[1]
ycdT	461	wt	7 nucleotide insertion TTTGTTT	as W3110	as W3110	as W3110	**Confirmed** (for 55989, HUSEC041 and LB226692)
yedQ	934	CAG (Q)	TAG (STOP)	TAG (STOP)	TAG (STOP)	as W3110	**Confirmed**
yegE	253	wt	as W3110	as W3110	1 nucleotide deletion (T)	as W3110	Disproven
yfgF	642	wt	as W3110	1 nucleotide deletion (A)	as W3110	as W3110	Disproven
yoaD	697	wt	as W3110	as W3110	1 nucleotide deletion (G)	as W3110	Disproven
yddV	352	wt	as W3110	as W3110	1 nucleotide deletion (T)	as W3110	Disproven
yfiN	155	wt	as W3110	as W3110	1 nucleotide deletion (A)	as W3110	Disproven
yddU	45	wt	as W3110	1 nucleotide deletion (T)	as W3110	as W3110	Disproven
yddU	1212	wt (AGT after nucl. 1212)	as W3110	as W3110	as W3110	10 additional nucleotides (CGTGGTGT ATCTC instead of AGT after nucl. 1212)	**Confirmed**
yeaI	3	ATG/Start	as W3110	as W3110	as W3110	ATT (I)	**Confirmed**
yhjH	489	wt	as W3110	as W3110	1 nucleotide deletion (G)	as W3110	Disproven
ycgF	58	Wt	as W3110	as W3110	1 nucleotide deletion (A)	*ycgF* is not present	Disproven (1-nucl. deletion in LB226692)

[1]All variations indicated by the reported genome sequences were controlled by sequencing of PCR fragments obtained with chromosomal DNA of the respective strains as a template. All of the one-nucleotide deletions indicated by the reported genome sequences were located in regions of stretches of the same nucleotide and were found to be due to sequencing errors ('Disproven').

Supplementary Table S1. SNPs and other small sequence variations in the coding regions of GGDEF/EAL domain-encoding genes in the outbreak O104:H4 strain (LB226692) in comparison to 55989, HUSEC041, EDL933 and W3110. Only genome sequence variations with clear consequences for gene product expression, such as small insertions or deletions as well as newly generated stop codons were taken into account (but not occasional single nucleotide variations that result in synonymous codons or conservative amino acid exchanges). GGDEF/EAL genes altered in LB226692 (as well as its close relatives 55989 and HUSEC041) are highlighted in red, those altered in EDL933 are highlighted in blue.

The TAG stop codon in ***yedQ*** is closely followed by an ATG codon (TAGGCAATG), which (in cases where translation stops, i.e. no readthrough occurs) would probably allow an efficient restart of translation due to translational coupling. As a result a N-terminally truncated YedQ protein with nevertheless intact GGDEF domain (which is encoded downstream of approximately nucleotide 1200) would be expressed.

The consequences of the alterations of *yddU* and *yeaI* as well as the absence of *ycgF* in

EDL933 are beyond the scope of the present study and have not been analyzed further.
