# Supplementary material for: Cyclic-di-GMP signalling and biofilm-related properties of the Shiga toxin-producing 2011 German outbreak Escherichia coli O104:H4
Source: EMBO Mol Med. 2014 Oct 31;6(12):1622–37. doi: 10.15252/emmm.201404309 (PMC4287979; doi:10.15252/emmm.201404309)
Supplement: Supplementary file 8 — Supplementary Table S2 [file emmm0006-1622-sd8.pdf]

Gene	Codon	W3110	55989	HUSEC041	LB226692	Protein function
**mutT**	89	CGC (R)	AGC (S)	AGC (S)	AGC (S)	8-oxo-(d)GTP hydrolase
**mutS**	337	GGG (G)	GAG (E)	GAG (E)	GAG (E)	Methyl-directed mismatch repair
**mutL**	83	CTG (L)	CAG (Q)	CAG (Q)	CAG (Q)	Methyl-directed mismatch repair
	389	CCC (L)	as W3110	as W3110	as W3110*	
	418	TTA (L)	CCA (P)	CCA (P)	CCA (P)	
**uvrD**		wt	as W3110	as W3110	as W3110	Helicase II, nucleotide excision repair
**mutD**	222	ATT (I)	CTT (L)	CTT (L)	CTT (L)	Subunit of DNA polymerase III
	233	CAG (Q)	GAG (E)	GAG (E)	GAG (E)	
**mutY**	217	GCG (A)	TCG (S)	TCG (S)	TCG (S)	Adenine glycosylase, mismatch repair
	272	CAG (Q)	CAT (H)	CAT (H)	CAT (H)	
**mutM**	94	GAT (D)	GAA (E)	GAA (E)	GAA (E)	DNA glycosylase in base excision repair
**dam**		wt	as W3110	as W3110	as W3110	DNA adenine methylase

Supplementary Table S2. **Putative mutations in the coding regions of genes for DNA maintenance and repair in the outbreak O104:H4 strain in comparison to 55989, HUSEC041 and W3110.** The genome sequences of the outbreak strain (LB226692) as well as its close relatives 55989 and HUSEC041 show a number of common amino acid variations in several known mutator genes in comparison to W3110. The reported genome sequence of LB226692 also features a deletion of one nucleotide in codon 389 of *mutL* (the last C in a row of 6 Cs; indicated by * in the table), suggesting a translational frameshift. However, resequencing this DNA region from a PCR fragment showed this variation to be a sequencing error.
