# Supplementary material for: Cyclic-di-GMP signalling and biofilm-related properties of the Shiga toxin-producing 2011 German outbreak Escherichia coli O104:H4
Source: EMBO Mol Med. 2014 Oct 31;6(12):1622–37. doi: 10.15252/emmm.201404309 (PMC4287979; doi:10.15252/emmm.201404309)
Supplement: Supplementary file 9 — Supplementary Table S3 [file emmm0006-1622-sd9.pdf]

**Supplementary Table S3. Oligonucleotide primers used in the present study.**

## I. Primers used for constructing *dgcX*::*lacZ* and *yneF*::*lacZ* on pJL28[1]:

| | |
|---|---|
| *dgcX*-d-412(*Eco*RI) | 5'- GCAAA**GAATTC**TGTGCCTCAGTTTTGTC-3' |
| *dgcX*-u-18(*Hin*dIII) | 5'- ATAG**AAGCTT**GGTTGATAATCATGTACGCACC-3' |
| *yneF*-d-373(*Eco*RI) | 5'- GGCG**GAATTC**GTCGCTAAACGTC-3' |
| *yneF*-u-14(*Hin*dIII) | 5'- GGGT**AAGCTT**GCATTAATTATCTGTCCATAC-3' |

## II. Primers used for PCR analysis of the *pgaA-ycdT* and *dgcX* regions in the chromosome:

| | |
|---|---|
| *pgaA*-E1(-2890)-for- | 5'-CAGGGTTAAAATTTGTCC-3' |
| *ycdT*-E2(+1446)-rev | 5'-CCTCAGTCCGGAACAATT-3' |
| *dgcX*-d-(-62) | 5'-GTAAGGAATTCTCGTTATTTGTAGCGATACATATTA-3' |
| *dgcX*-u-1310 | 5'-AAAAGGAGATCTTTTTGAGTGAGTTATCACC-3' |

## III. Primers used for the construction of insertion-deletion mutations in *bcsE, bcsF* and *bcsG*[2]:

| | |
|---|---|
| *bcsE*-H1P1-pKD13 | 5'-GATAAGTTTTAATTTCAATGGTAGGTTTATTTCTTAGCTTTCGCTAG*GTGTAGGCTGGAGCTGCTTC*-3' |
| *bcsE*-H2P4-pKD13 | 5'-GCCAGTGGCGACCATCGTGCTCGGCATTGATGACCGGTTTAGAACTTTGC*ATTCCGGGGATCCGTCGACC*-3' |
| *bcsF*-H1P1-pKD13 | 5'-GAAATTATTGTCGTTTGCGCACTGATATTTTTCCCGCTGGGCTATCTGGC*GTGTAGGCTGGAGCTGCTTC*-3' |
| *bcsF*-H2P4-pKD13 | 5'-CCGTGCGGCGTAACGTCCCGGCCGGTTTAACATAACGAGGTTTAGCAAAG*ATTCCGGGGATCCGTCGACC*-3' |
| *bcsG*-H1P1-pKD13 | 5'-GGCGCGGCCTTTCCGGCTGGAACTTCTATTTTCTGGTTAAGTTCGGCCTG*GTGTAGGCTGGAGCTGCTTC*-3' |
| *bcsG*-H2P4-pKD13 | 5'-TTACTGCGGGTAAGGCACCCAGTCGCCGCCGTTCAGGCGAACGTACGG*ATTCCGGGGATCCGTCGACC*-3' |

## IV. Primers used for cloning into *csgD* into pQE60[3]:

| | |
|---|---|
| *csgD*-*Nco*I | 5'-TTCATG**CCATGG**TTAATGAAGTCCATAGTATTCATGGTC-3' |
| *csgD*-*Bgl*II | 5'-AATGGA**AGATCT**TCGCCTGAGGTTATCGTTTGCCCAGGAAACC-3' |

[1] Relevant restriction sites are given in **bold**.

[2] Sequences complementary to pKD13 (providing the resistance cassette) are given in *italics*.

[3] Relevant restriction sites are given in **bold**.
