# Supplementary material for: Cyclic-di-GMP signalling and biofilm-related properties of the Shiga toxin-producing 2011 German outbreak Escherichia coli O104:H4
Source: EMBO Mol Med. 2014 Oct 31;6(12):1622–37. doi: 10.15252/emmm.201404309 (PMC4287979; doi:10.15252/emmm.201404309)
Supplement: Supplementary file 10 — Legends for Supplementary Figures [file emmm0006-1622-sd10.pdf]

LEGENDS TO SUPPLEMENTARY FIGURES

Supplementary Figure S1. Amino acid sequence alignment of DgcX with its most closely related GGDEF domain proteins in *E. coli,* YeaI and YcdT. The alignment was generated using CLUSTAL-X (Thompson et al. 1997. Nucl. Acids Res. 25: 4876-4882) and manually colour-edited to highlight key amino acid positions associated with DGC activity.

Supplementary Figure S2. A comparison of the chromosomal *pgaA-ycdT* region in *E. coli* K-12 (W3110), the O104:H4 outbreak strain, HUSEC041 and EAEC 55989.

A. Different lengths of the *pgaA-ycdT* region and the presence or absence of the *dgcX* region in W3110, the outbreak strain (isolates LB55989 and RKI II-2027), HUSEC041 and EAEC 55989 as detected by agarose electrophoresis of PCR fragments obtained with chromosomal DNAs as templates (primers are listed in Supplementary Table S3).

B. Promoter sequences upstream of *pgaA*. The original *pgaA* promoter sequence as present in W3110 as well as in the outbreak strain LB226692 and HUSEC041, its spacer length and transcriptional start site in W3110 are highlighted in red. Specifically in EAEC 55989, the upstream part of the *pgaA* promoter region is replaced by an insertion of IS1 (sequence underlied in yellow). Two alternative promoter sequences that have been created by the IS1 insertion in strain 55989 are highlighted in blue and red. 5´-UTR, 5´-untranslated region of the mRNAs generated.

Supplementary Figure S3. Expression of *lacZ* reporter fusions to *dgcX* and *yneF* during the growth cycle.

A: Derivatives of strain W3110 (circles) and an otherwise isogenic *rpoS* mutant (squares) carrying a single copy *dgcX*::*lacZ* fusion (relevant sequence obtained from the O104:H4 outbreak strain) were grown in LB at 28ºC. Optical densities (open symbols) and specific ß-galactosidase activities (closed symbols) were determined.

B: Similar experiment as in (A), but with strains carrying a single copy *yneF*::*lacZ* fusion.

Supplementary Figure S4. Macrocolony morphology of the outbreak O104:H4 strain in comparison to 55989, HUSEC041, EDL933 and W3110 grown at different temperatures and salt concentrations. The strains were spotted onto LB plates with (LB) or without salt (LBnoS) and grown at the indicated temperatures for seven days.

Supplementary Figure S5. The *stx2*-carrying CP-933V prophage in EDL933 is inserted into the *mlrA* gene. The prophage CP-933V (indicated by a box in the figure of the EDL933 chromosome) is inserted upstream of codon 29 in the *mlrA* gene (formerly *yehV*), i.e. the promoter and translational start regions as well as the first 28 codons of *mlrA* are missing. Codon 29 (TTG) has been annotated as a start codon in the genome sequence of EDL933. However, even in the unlikely case that translation may start there (no ribosomal binding site is apparent in the sequence), it would lead to a N-terminally truncated MlrA, in which approximately half of the N-terminal DNA-binding domain would be missing. As a consequence, EDL933 does not produce CsgD, curli and cellulose and grows in flat and unstructured macrocolonies.

Supplementary Figure S6. The *bcsE* gene in the O104:H4 outbreak strain and the role of the *bcsEFG* operon in cellulose production.

A. Genomic arrangement of the two *bcs* operons involved in cellulose biosynthesis. The presence of a frameshift mutation results in premature termination of BcsE synthesis in the O104:H4 outbreak strain as indicated by a broken arrow.

B. Introducing non-polar deletion mutations in *bcsE*, *bcsF* and *bcsG* into strain AR3110, a curli^{+} cellulose^{+} derivative of strain W3110, alters colony morphology from a elaborately wrinkled to a concentric ring pattern characteristic of the production of curli alone (compare to Fig. 4), indicating that each gene in the *bcsEFG* operon is required for cellulose biosynthesis.
