# Supplementary material for: Cyclic-di-GMP signalling and biofilm-related properties of the Shiga toxin-producing 2011 German outbreak Escherichia coli O104:H4
Source: EMBO Mol Med. 2014 Oct 31;6(12):1622–37. doi: 10.15252/emmm.201404309 (PMC4287979; doi:10.15252/emmm.201404309)
Supplement: Supplementary file 1 — Supplementary Figure S1 [file emmm0006-1622-sd1.pdf]

```
DgcX          ------------------------------------------MIINQVPIKIK---IFI  23
YeaI          MIQSTRISMGLFFKYFLSLTKIDPGQNYISLPSIKSSTHIALLFMVSMGTQKLKAQSFFI  60
YcdT          ---------------------------------------------MEKDYLRIS--STVL  13
                                                           :.    ::.    .:

DgcX          FLFSCISIIFLLLHANNG-IYITQTTQISYSVFIIGLFFINLMIFIFLLLYYVSNQRQSY  73
YeaI          FSLLLTLILFCITTLYNENTNVKLIPQMNYLMVVVALFFLNAVIFLFMLMKYFTNKQILP 120
YcdT          VSLLFGLALVLVNSWFNQPGVEEVVPRSTYLMVMIALFFIDTVAFIFMQLYFIYDRRQFS 73
              . :     :. :    *        .: .* :.::.***:: : *:*: : :. :::

DgcX          LLILSFAFLSNTYYLLEVAIISLSPLGNDLSTIYQKSNDIAIYYLFRQFSFISIIFLAVY 133
YeaI          TLILSLAFLSGLIYLVETIVIIHKPIN-GSTLIQTKSNDVSIFYIFRQLSFICLTSLALF 179
YcdT          NCVLSLAFLSCLIYFVITVIIIQQIIEERLTSSVVQ-NDIAIYYLFRQMSLCILIFLALV 132
                :**:****   *:: . :*  . :    :    : **::*:*:***:*:  :  **:

DgcX          STNVKNKSVLED-KRNIIIVVLSILILFITPFVAKNLSSDNIKYSLNIIQYSLNRHLPTW 192
YeaI          CYGKDN--ILDNNKKKTGILLLALIPFLVFPLLAHNLSSYNADYSLYVVDYCPDNHTATW 237
YcdT          NKVSEN-TKQRNLFSKKMTLCISLFFVFGGPIVAHILSSHYESYNLHIAELTNENGQVVW 191
                  .*     :   :   : :::: .:  *::*: ***   .*.* : :   :.   .*

DgcX          NIVYTKIISVFWLVLLISSCISIRNYSKIWLCIILISIVSVCNNLILLYFIDKSHPAWYM 252
YeaI          GINYTKILVCLWAFLLFFIIMRTRLASELWPLIALLCLASLCCNLLLLTLDEYNYTIWYI 297
YcdT          KASYVTIMIFMWLTLLSVNLYFNGLRYDIWNGVTVIAFCAVLYNISLLFMSRYSVSTWYI 251
                 *..*:  :*  **           .:*  : ::.: ::  *: ** :   . . **:

DgcX          TKFLELISMIYIISTLMYYIFRKLNHANHMAIHDPLTNTYNRRYFIDSLKNISKH---HD 309
YeaI          SRGIEVSSKLFVVSFLIYNIFQELQLSSKLAVHDVLTNIYNRRYFFNSVESLLSRPVVKD 357
YcdT          SRTIEVVSKLTVMVIFMCHIFSALRVTKNIAHRDPLTNIFNRNYFFNELTVQSASAQKTP 311
              :: :*: * : ::  ::  **  *. :.::* :* *** :**.**::.:

DgcX          FSVIMLDIDSFKSINDKWGHHMGDQVIVMVTRIIKKSIRKEDILGRLGGEEFGIIIKGNT 369
YeaI          FCVMLVDINQFKRINAQWGHRVGDKVLVSIVDIIQQSIRPDDILARLEGEVFGLLFTELN 417
YcdT          YCVMIMDIDHFKKVNDTWGHPVGDQVIKTVVNIIGKSIRPDDLLARVGGEEFGVLLTDID 371
              :.*:::**: ** :*  *** :**:*:  :. ** :*** :*:*.*: ** **:::.

DgcX          QKLLLSIAERIRKNIEEQCSEKLLSHGPEKITVSIGCFTSKENNLSPSEMLVNADKALYQ 429
YeaI          SAQAKIIAERMRKNVELLTGFSNRYDVPEQMTISIGTVFSTGDTRNISLVMTEADKALRE 477
YcdT          TERAKALAERIRENVERLTGDNPEYAIPQKVTISIGAVVTQENALNPNEIYRLADNALYE 431
                    :***:*:*:*   . .     *:::*:*** . :  :  . . :   **:** :

DgcX          AKRTGKNKVIIHSK-------- 443
YeaI          AKSEGGNKVIIHHI-------- 491
YcdT          AKETGRNKVVVRDVVNFCESP 452
              **  * ***:::
```

A-site   I-site
